# Effect of the COVID-19 pandemic on cataract surgery by residents who had routine surgical simulator training during residency

**DOI:** 10.5935/0004-2749.2023-0038

**Published:** 2024-03-05

**Authors:** Maria Clara O. Magalhães, Mariana M. G. Sarmento, Guilherme H. Sant’Anna, Ana Karine A. Soares, Camila V. Ventura, Camilla S. Rocha, Bruna V. Ventura

**Affiliations:** 1 Department of Ophthalmology, Fundação Altino Ventura, Recife, PE, Brazil; 2 Department of Research, Fundação Altino Ventura, Recife, PE, Brazil; 3 Hospital de Olhos de Pernambuco, Recife, PE, Brazil

**Keywords:** COVID-19, Pandemics, Cataract extraction/education, Internship and residency/methods, Simulation training/methods, Phacoemulsification/education, Surgery, computer-assisted, Computer simulation, Clinical competence, Ophthalmology/education

## Abstract

**Purpose:**

To assess the effect of the Coronavirus disease 2019 (COVID-19) pandemic on cataract surgery by residents who had mandatory surgical simulator training during residency.

**Methods:**

In this retrospective, observational analytical study, the total number of cataract surgeries and surgical complications by all senior residents of 2019 (2019 class; prepandemic) and 2020 (2020 class; affected by the reduced number of elective surgeries due to the COVID-19 pandemic) were collected and compared. All residents had routine mandatory cataract surgery training on a virtual surgical simulator during residency. The total score obtained by these residents on cataract challenges of the surgical simulator was also evaluated.

**Results:**

The 2020 and 2019 classes performed 1275 and 2561 cataract surgeries, respectively. This revealed a reduction of 50.2% in the total number of procedures performed by the 2020 class because of the pandemic. The incidence of surgical complications was not statistically different between the two groups (4.2% in the 2019 class and 4.9% in the 2020 class; p=0.314). Both groups also did not differ in their mean scores on the simulator’s cataract challenges (p<0.696).

**Conclusion:**

Despite the reduction of 50.2% in the total number of cataract surgeries performed by senior residents of 2020 during the COVID-19 pandemic, the incidence of surgical complications did not increase. This suggests that surgical simulator training during residency mitigated the negative effects of the reduced surgical volume during the pandemic.

## INTRODUCTION

In March 2020, the World Health Organization classified the Coronavirus disease 2019 (COVID-19) outbreak as a pandemic. In many countries, elective procedures were suspended for months. One of the most affected specialties was ophthalmology, and many centers were converted into COVID-19 wards and reported a decrease in >50% of their general surgical volume^(1,2,3)^. Cataract surgery is one of the most performed surgical procedures worldwide. However, since it is an elective surgery in most cases, it was greatly affected by the COVID-19 pandemic^(1,2,3)^. In this scenario, ophthalmology teaching institutions faced the challenge of ensuring that their residents develop cataract surgical skills despite the drawbacks of the pandemic.

Cataract surgery is a step-dependent complex procedure that demands good stereoacuity, excellent hand-eye coordination, and ability to use all four limbs simultaneously^(4)^. These competencies are acquired with training^(5,6)^. Previous studies have shown that a minimum of approximately 75-80 surgeries are necessary for residents to perform straightforward cataract cases without assistance. However, surgical competency continues to improve with increasing surgical experience, reflecting a reduction in complication rates^(5,6,7)^.

An interruption in phacoemulsification training for a few lockdown months, associated with a general reduction in the annual cataract surgeries performed in 2020, could potentially have affected the surgical skills of third-year ophthalmology residents, increasing the complication rates. Since our residents have mandatory training using the Eyesi surgical simulator (VRmagic GmbH) as part of their residency program and many studies have shown its important role in the learning curve in a regular scenario^(8,9,10,11,12,13)^, this study aimed to assess the effect of the COVID-19 pandemic on cataract surgeries and surgical performance of our senior residents.

## METHODS

This retrospective observational analytical study evaluated the total number of cataract surgeries and surgical complications of all third-year ophthalmology residents of the Altino Ventura Foundation, in Recife, Brazil, in 2019 (2019 class) and 2020 (2020 class). The study protocol followed the guidelines of the Declaration of Helsinki and was approved by the Ethics Committee of the Altino Ventura Foundation (IRB Protocol No/FAV: 3.912.877).

The 2019 class finished their residency before the pandemic started. The 2020 class was affected by a 2-month lockdown at the beginning of their cataract surgery training in the operating room (April and May 2020). During these months, no elective surgeries were performed in the state of Pernambuco. In June 2020, elective surgeries were allowed to be scheduled and performed starting with minimal numbers owing to the need for social distancing and slowly increasing throughout the year.

The total number of cataract surgeries and surgical complications per resident were collected from the electronic registry of the operating rooms. For the present study, surgical complications encompass bag dialysis and capsular tear with or without vitreous loss.

The Eyesi is a virtual surgical simulator that reproduces all steps of cataract surgery, generating objective measures that translate residents’ surgical proficiency^(8,10,11)^. After every hour of training, the resident is exposed to a cataract challenge, which consists of an entire simulated cataract surgery, including capsulorhexis confection, hydrodissection and hydrodelineation, phacoemulsification, cortical aspiration, and intraocular lens implantation. The maximum score obtained in this activity is 500 points. In this study, the scores of each resident on all cataract challenges were collected from the simulator’s online registry and used to assess the residents’ surgical performance on the simulator.

The Altino Ventura Foundation received its surgical simulator in June 2019 (Eyesi surgical simulator, 2019, serial # 378). From then on, it became part of all residents’ monthly rotation. Thus, the 2019 class had routine mandatory training in the surgical simulator during half of their third year of residency, whereas the 2020 class started this training when they were halfway through their second year of residency. During the 2-month lockdown, the 2020 class had no elective surgeries or simulator exposure because the government regulation allowed only essential medical activities.

### Statistical analysis

Data analysis was performed using IBM SPSS Statistics version 25.0 (IBM Corp., Armonk, NY, USA). Quantitative variables are expressed as mean, standard deviation, minimum, and maximum values. Qualitative variables are expressed as absolute and relative frequencies. Normality was checked using the Shapiro-Wilk test. The Student t-test was used to compare the number of surgeries, complication rates, and simulator score between the two classes, whereas the Mann-Whitney test was used when the variables were not normally distributed. A p<0.05 was considered statistically significant.

## RESULTS

A total of 37 residents participated in the study: 20 (54.1%) from the 2019 class and 17 (45.9%) from the 2020 class. The 2019 class had a mean total training time in the surgical simulator of 11:35 ± 0.1 h (range, 07:45-18:14), whereas the 2020 class had a mean of 18:43 ± 0.2 h (range, 07:07-28:00) of training (p<0.001). The 2019 class performed 2561 cataract surgeries, whereas the 2020 class performed a total of 1275 surgeries, evidencing a decrease of 50.2% in cataract surgical volume (1286 surgeries) during the pandemic. The mean number of procedures per resident was 128.1 ± 20.0 (range, 90-169) in the 2019 class and 75.0 ± 17.0 (range, 50-111) in the 2020 class (p<0.001).

**Figure 1:**
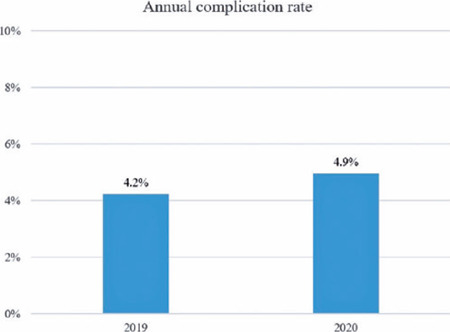
Annual complication rate by senior residents during the pre-pandemic year (2019 class) and COVID-19 pandemic year (2020 class).

The total number of surgical complications was 108 and 63 in the 2019 class and 2020 class, which corresponded to an annual complication rate of 4.2% and 4.9% (p=0.314), respectively (Figure 1). The mean number of complications per resident was 5.4 ± 2.1 (range, 0-9) in the 2019 class and 3.7 ± 1.8 (range, 0-7) in the 2020 class (p<0.015).

**Figure 2:**
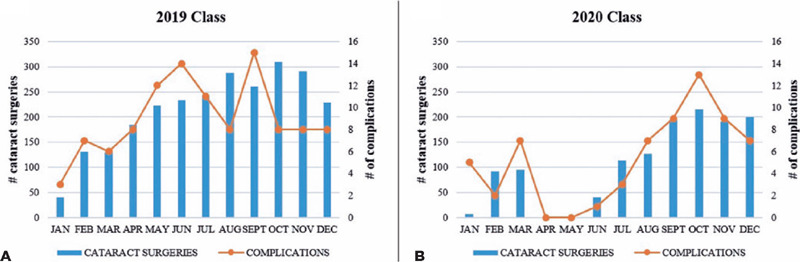
Monthly number of cataract surgeries and complications by senior residents during the (A) prepandemic year (2019 class) and (B) pandemic year (2020 class).

Figure 2 presents the number of cataract surgeries and complications per month per class.

The mean score on the simulator’s cataract challenge was 383.3 ± 40.8 (range, 305.7-460.3) points in the 2019 class and 378.85 ± 24.8 (range, 319.5-408.9) points in the 2020 class (p<0.696).

## DISCUSSION

The COVID-19 pandemic had a huge effect on ophthalmology institutions, with a decrease in the number of consultations and surgeries. For a period, all elective procedures were suspended in many countries, which interrupted the learning curve of cataract surgery by residents, followed by a period of a reduced number of scheduled procedures^(1,2,3)^. This could potentially have a negative effect on their training, resulting in more surgical complications. Since several studies have shown the benefit of virtual surgical simulator training during residency^(8,9,10,11,12,13,14,15,16,17,18)^ and our residents have routine training on it, in the present study, we assessed how the pandemic affected cataract surgery and surgical performance among our 2020 senior residents.

The pandemic resulted in a 50.2% decrease in cataract surgeries performed by our third-year residents in 2020 when compared with the senior residents in 2019. Similar to what was seen in other parts of the world^(1,2,3)^, in our state, we had a lockdown period in which no elective surgeries were scheduled for 2 months, followed by several months in which a reduced number of elective procedures were performed. Although our senior residents in 2020 had been routinely exposed to mandatory virtual reality cataract surgical training since their second year of residency, the lockdown occurred after only a few months of them actually starting to perform phacoemulsification in patients. Thus, this early interruption in their operating room learning curve, associated with a reduction in half of the number of total cataract surgeries performed in their third year of residency, could potentially have a huge effect on their surgical competency, which may be translated into complication rates^(6,7)^.

Several studies have tried to determine a minimum number of cataract procedures that a resident should perform throughout residency to perform routine cataract surgery without increased risk of intervention or complications^(5,6,7)^. Taravella et al.^(7)^ suggested a minimum of 75 surgeries, whereas Randleman et al.^(6)^ reported that an increase in surgical efficiency was associated with a decrease in complication rates after 80 procedures. Even though our third-year residents in the 2020 class had performed cataract surgery in a mean of 75 patients, achieving the guidelines suggested by these two authors, improvements in surgical efficiency and reduction in complication rates continue beyond the resident’s first 200 cases without reaching a plateau^(6,19)^. Thus, as the 2019 class had almost double the surgical volume of the 2020 class and their third-year surgical training was not interrupted, they would have a statistically lower mean complication rate than the 2020 class.

Interestingly, the 2020 class presented an annual complication rate statistically similar to the 2019 class. Furthermore, no statistically significant difference was found in the simulator’s cataract challenge mean score between the two groups, suggesting that they had comparable virtual surgical proficiencies. As reported by Thomsen et al.^(20)^, Bozkurt Oflaz et al.^(11)^, and Jacobsen et al.^(8)^, performance on the surgical simulator correlates with real-life surgical performance. In addition, many studies have shown the beneficial effect of regular simulator training on reducing complication rates, surgical time, phacoemulsification time, and learning curve^(4,17,18)^. Thus, we postulate that the comparable complication rates between the two groups, despite the difference in the surgical volume and all drawbacks associated with the pandemic, is attributed to the positive effect of the routine surgical simulator training in the cataract surgery learning curve of our residents.

This study is mainly limited by its retrospective nature and all associated disadvantages. In addition, both groups of senior residents did not have the same amount of simulator training during residency. However, all residents concluded all cataract activities offered by the simulator training and then continued using the simulator to improve their proficiency in specific steps they judged necessary.

The COVID-19 pandemic resulted in a 50.2% decrease in the surgical volume of our senior residents in 2020. However, this was not accompanied by an increase in complication rates when compared with the third-year residents of 2019. We postulate that the surgical simulator routine training during residency mitigated the negative effect of the reduced surgical volume during the pandemic.
